# Expanding the role of the future zoo: Wellbeing should become the fifth aim for modern zoos

**DOI:** 10.3389/fpsyg.2022.1018722

**Published:** 2022-10-20

**Authors:** Paul E. Rose, Lisa M. Riley

**Affiliations:** ^1^Centre for Research in Animal Behaviour, Psychology, University of Exeter, Exeter, United Kingdom; ^2^WWT, Slimbridge Wetland Centre, Gloucestershire, United Kingdom; ^3^Centre for Animal Welfare, University of Winchester, Winchester, United Kingdom

**Keywords:** wellbeing, zoo aims, conservation, engagement, connection with nature, one health, one welfare

## Abstract

Zoos and aquariums have an enormous global reach and hence an ability to craft meaningful conservation action for threatened species, implement educational strategies to encourage human engagement, development and behavior change, and conduct scientific research to enhance the husbandry, roles and impacts of the living collection. The recreational role of the zoo is also vast- people enjoy visiting the zoo and this is often a shared experience amongst family and friends. Evaluating how the zoo influences this “captive audience” and extending its reach to include a compassionate approach to animals and people can further enhance the mission, value, and relevance of their work. The modern zoo’s current aims—Conservation, Education, Research and Recreation—provide useful foundations for the activities that zoos conduct at a local and national level. However, to improve sustainability of their actions and outcomes, we feel that Wellbeing should become the fifth aim of the modern zoo for the future- both from an animal perspective (ensuring that populations are managed according to prevailing behavioral needs) and from a human perspective (enhancing access to nature, promoting planetary friendly behavior changes and ways of living, and advancing the wellbeing of the zoo’s workforce). This paper provides discussion and review of how Wellbeing is already a substantial part of what zoos work on as well as posing the idea of altering the Recreation aim of the zoo to one of Engagement, which potentially is more measurable and therefore can allow zoo researchers and managers further options for the collection of evidence on the local and global reach of their zoo’s aims. Education, Engagement, Conservation, Research and Wellbeing provide a more complete picture of the roles of the modern zoo for the animals (both *in situ* and *ex situ*), human visitors and workforce, and to society more widely.

## Introduction

With an estimated reach of 700 million annual visitors (Zordan, 2021), the global collective of zoos and aquariums (hereafter “zoos”) are in a unique position to promote environmental awareness across vast numbers of people. Developing “planetary friendly” behavior change, ensuring biodiversity conservation and crafting a deeper connection with the natural world supports UN Sustainability Goals (United Nations, 2021). Such activities are also important to long term human physical and psychological health. Accredited / member zoos (i.e., those that are part of zoo membership organizations such as AZA or EAZA and are inspected against the standards of such organizations) are also well-placed to help enable the four goals of the Post-2020 Global Biodiversity Framework, with its specific vision of *“By 2050, biodiversity is valued, conserved, restored and wisely used, maintaining ecosystem services, sustaining a healthy planet and delivering benefits essential for all people”* (Secretariat of the Convention on Biological Diversity, 2021). Given that individual zoos can garner considerable funding for conservation and be involved in numerous global, multi-stakeholder conservation projects (Maynard et al., 2020), marrying up this “conservation power” with huge visitor appeal provides an easy way of spreading biodiversity and sustainability messaging.

To encapsulate their wide influence, since the 1980s the modern zoo’s activities centered around four main aims of Conservation, Education, Recreation and Research (CERR; Kleiman, 1985). The species managed in the zoo’s living collection provide support to how the zoo meets these aims (Rose, 2018) and are excellent proxies for wider activities with global impact (Clayton and Le Nguyen, 2018). For example, zoo-housed species enable the implementation of effective conservation action (Kerr, 2021) or help to measure the influence of conservation education (Patrick et al., 2007). Several texts expand on the prominence of those goals to the zoo’s justification of its existence and of the work that it intends to accomplish, e.g., the balance between conservation and education against tourism (i.e., entertainment; Frost, 2011) or a shift in focus to research and conservation at the heart of the zoo’s motives (Fraser and Wharton, 2007).

The zoo as a concept is always going to be a controversial one (Wickins-Dražilová, 2006; Maynard, 2018). Animals can appear managed in restricted areas for human gain (Carr and Cohen, 2011), that interactions with visitors are not for the animal’s benefit (Normando et al., 2018) or that the zoo’s conservation ideals are not always fully met by the presence of the animal collection (Arumugam et al., 2020). Species may not thrive in captive care due to specific facets of their natural history (Clubb and Mason, 2003; Mason, 2010) and stress responses can differ between free-living and captive individuals (Terio et al., 2004), especially when animals cannot remove themselves from human presence (Vasconcellos et al., 2016). Deviations in environmental conditions in captive, when compared to the wild, can result in long term poor health and reduced longevity (Potter and Clauss, 2005). To justify their positive attributes, it is therefore essential that zoos continue to strive with improvements to husbandry and management on a species-specific level (Melfi, 2009; Troxell-Smith and Miller, 2016), as well as continue to assess the needs of the individuals within these species that they house (Clay and Visseren-Hamakers, 2022).

This article evaluates why a further shift in the aims of the modern zoo should be to include wellbeing as a core reason for their existence. For the purposes of this paper, we define CERR as: Conservation- the management of populations for *ex situ* breeding and support for field-based recovery programs; Education-formalized learning sessions and explanation and interpretation of biodiversity and ecological messages; Research- output from basic and applied science pertaining to the zoo’s core mission and objectives; Recreation- provides an engaging and stimulating experience for visitors. These definitions are based on the key outputs that zoos strive to achieve as well as how they engage with their visitors, keepers and personnel, and the values provided within their mission statements (Patrick et al., 2007; Patrick and Caplow, 2018). Given the debate over how well zoo activities cover all of their mission statements, especially concerning conservation (Maynard et al., 2020), we feel that zoos should expand their scope to consider how they conserve animal welfare and engage with human wellbeing. We believe that the modern zoo should promote Wellbeing as its fifth core aim, with Wellbeing encompassing the welfare of the animals housed in the living collection and the wellbeing of the visitors that engage with the zoo’s mission by visiting the organization.

## Promoting connectivity with nature

Connection with nature is important to the maintenance of healthy human mental health (Bratman et al., 2012) and the concept of “green prescribing”—nature-based interventions and activities to restore positive mental states (National Health Service, 2022)—can help with the treatment of mental health conditions, such as anxiety and depression (White et al., 2021). The zoo’s Recreational aim may be one of the most accessible forms of green prescribing to urban populations that are seeking to connect with nature. Especially as research indicates that negative feelings of loneliness are significantly reduced when city-dwelling people engage with nature (Hammoud et al., 2021). Public aquariums and the nature reserves managed by zoos also provide a form of blue prescribing, promoting the value of wetlands to human quality of life and planetary health (i.e., holistic, sustainable, interconnected health for people, animals and the environment; Cracknell, 2016; Gearey et al., 2019; Reeves et al., 2021). Reduced responses to stress are noted in humans that experience blue spaces (Reeves et al., 2019), further supporting the Wellbeing aim of the blue spaces managed by zoos. Consciously or subconsciously, zoos set out to promote wellbeing and to enhance the quality of life of their visitors whilst at the same time, providing appropriate environments for their animals to experience positive welfare in.

**Figure 1:**
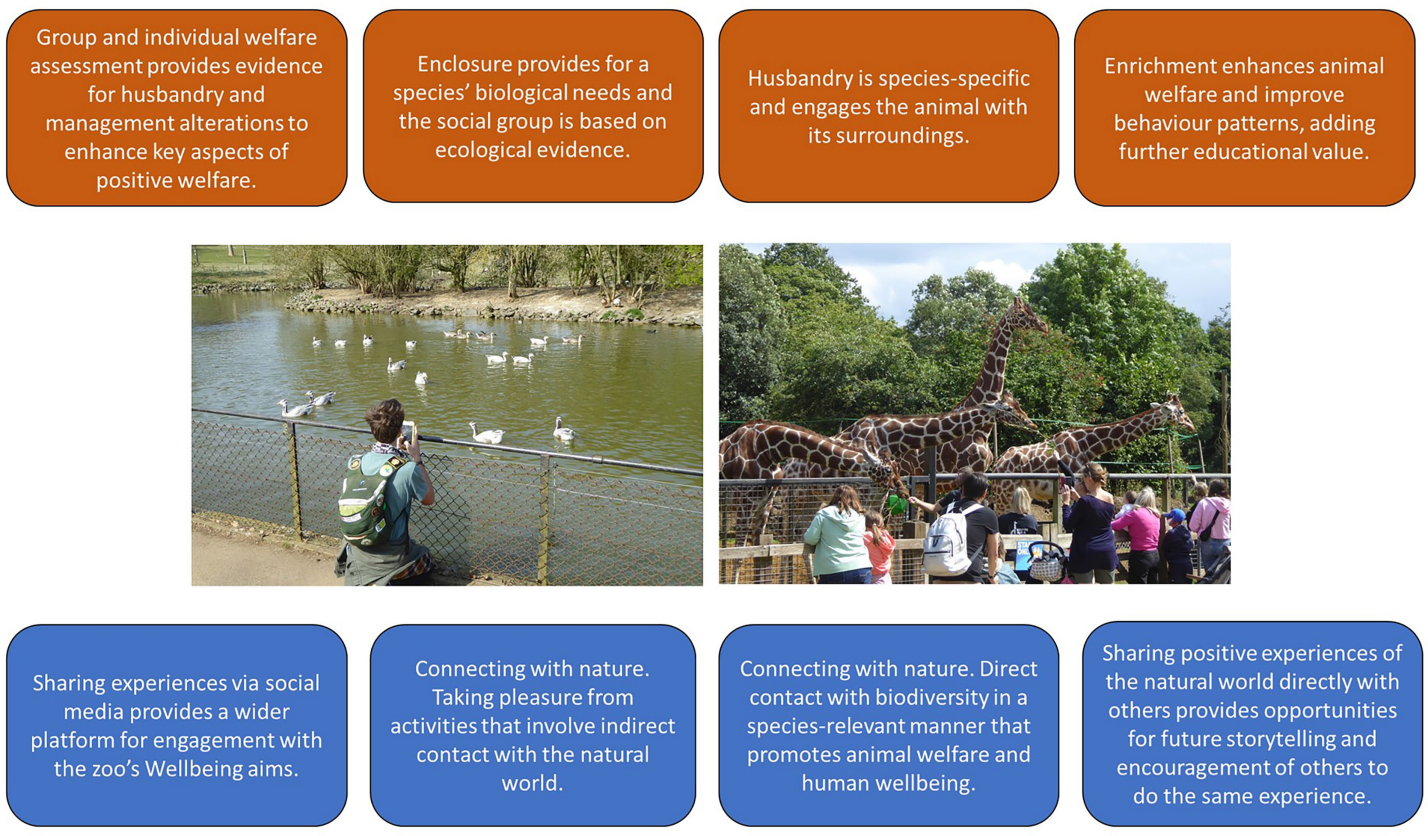
Wellbeing as a key aim of the modern zoo defined from the animal (brown boxes) and human (blue boxes) perspective. Animal welfare is embedded in all areas of the zoo’s operations pertaining to its living collection. Top left boxes: A biologically relevant social group and an enclosure that promotes adaptive behaviors enhances the chance of attaining positive welfare states. Bottom left boxes: Sharing a connection with nature and feeling connected to nature (that is enhanced when animals in the zoo look and act naturally) promotes positive human wellbeing. Top right boxes: Husbandry activities, such as use of enrichment or browse feeding to bring giraffes closer to visitors, is an ecologically relevant form of species care and increases the outward display of natural behaviors that further engage visitors. Bottom right boxes: A direct connection with nature occurs by sharing an experience of animal behavior at the zoo provides a lasting impact on the importance and value of biodiversity- one that can be used to impart ideas for human behavior change.

Key to this idea is that zoos provide tangible conservation of green and blue spaces—i.e. they are not likely to be developed and therefore will continue to provide access to nature—and this is especially true of those zoos in urban areas where access to green space may be restricted. The green spaces within zoos can be made accessible to large numbers of people with potential widespread health and wellbeing benefits. Direct contact with the natural world (and with animals in particular) also enhances positive mental states (Maller et al., 2006; Yerbury et al., 2021). Whilst access to many types of green space has consistently shown to promote many positive affective states in humans (Mensah et al., 2016), the green environment of the zoo that is combined with the animal collection may be more enhancing for (positive) human wellbeing than visits to other forms of green space (Akiyama et al., 2021). Being close to an animal has psychological benefits, which are further enhanced if an educator is also involved with the visitor-animal interaction (Sahlin et al., 2019). Therefore, including Wellbeing as the fifth aim of the modern zoo places an emphasis on the usefulness and importance of zoo green and blue spaces to their visitors and the local community, and shows how these spaces can be used to further engage zoo visitors in a deeper consideration and appreciation of the natural world (Figure 1).

Social media is a powerful tool for the wider dissemination of information that emphasizes the importance of engagement with biodiversity conservation and welfare in the zoo (Light and Cerrone, 2018; Rose et al., 2018; Llewellyn and Rose, 2021). Targeted and thoughtful use of social media can promote wider conservation objectives to audiences that may otherwise be hard to reach, and promote access to biodiversity/conservation education information in a more accessible manner (Bezanson et al., 2022). There may be added wellbeing benefits from such indirect connection to nature that the sharing of positive zoo content on social media platforms can bring to online audiences (i.e., bringing examples of the natural world closer to people whilst they go about their daily lives, and then giving them the idea to directly visit nature at the zoo). This further extends the reach of the zoo and how it can influence the wellbeing of human “visitors.” As non-zoo visitors are more likely to perceive zoo animals as experiencing poor welfare (Reade and Waran, 1996), indirect engagement *via* social media posts on species-typical behaviors and ecologically relevant exhibits can promote the good work of zoos to a skeptical audience. Alongside of the direct contact with nature that the zoo provides, relevant use of social media could be useful in creating opportunities for beneficial engagement, for sharing ideas to improve animal welfare with other institutions (e.g., enrichment ideas that can be noted from non-scientific literature) and therefore for supporting the Wellbeing aim of the zoo for animal and human benefits.

## Benefits to humans and animals

As sentient beings [albeit on a sliding scale of consciousness (Dawkins, 2022)] captive wild animals should experience good welfare and “a good life” (Mellor, 2016), i.e., one where the balance of positive experiences outweighs the negative. Humans have a moral, ethical and (in many countries) a legal obligation to ensure that the welfare of captive wild animals is good. Animals are invested in living a life they have evolved to live given their ecological niche and behavioral biology. Humans must therefore provide every plausible opportunity for captive wild animals to experience good welfare, from enclosure design to nutrition, enrichment, and veterinary care. Good welfare is of inherent value to each captive animal. Mason et al. (2007) argue that zoos need to have a “zero tolerance approach” to abnormal behaviors that can indicate negative welfare states, and this is an important consideration to any future Wellbeing aim. As societal attitudes become more critical of poor welfare, and zoo visitors more knowledgeable around the signs of poor welfare, so indicators of poorer welfare are likely to become more identifiable to more people.

Good animal welfare is also essential if visitors are to leave the zoo feeling empathetic to the zoo’s messages, if they wish to return for multiple visits and be invested in the zoo’s mission post-visit (Miller, 2012; Ballantyne and Packer, 2016; Minarchek et al., 2021). As zoo animals can display highly visible abnormal behavior patterns that can be used to infer poor welfare (Rose et al., 2017), regular evaluation of husbandry and management is required at the individual, species and population level to ensure that welfare challenges are resolved (Mason, 2010). Changes to policy that regulate zoo operations nationally and internationally is placing animal welfare as a key consideration when captive wild species are managed by humans. Healthy animals, performing species appropriate behaviors are more likely to have a positive influence on zoo visitors (Miller, 2012; Godinez et al., 2013; Sampaio et al., 2021) and this is supportive of the zoo’s key educational and engagement outputs. As different zoo-housed species elicit different emotional responses in visitors, and such emotional responses can be overwhelmingly positive (Myers et al., 2004), any perception of animal welfare is important for developing positive emotional states in zoo visitors.

The performance of species appropriate behavior, including opportunities to express natural behaviors with an adaptive function, enhances the relevance of captive individuals to conservation work (Buchholz, 2007; Martin-Wintle et al., 2015). Animals that are psychologically and physically fit are better candidates for breeding programs, research subjects and as tools for educational messaging that explain such conservation objectives (Hacker and Miller, 2016; Prescott and Lidster, 2017; Greggor et al., 2018). Ensuring that zoo-housed animals are managed according to species-appropriate evidence enhances how the zoo meets its aims (Rose et al., 2019; Rose, 2021) and all zoos should ensure that they are engaging with the latest evidence on correct species’ care to remain current and relevant (Melfi, 2009; Rose et al., 2019). Just as animals have to respond to a changing world—which emphasizes a need for building behavioral flexibility and resilience by use of suitable environmental enrichment and husbandry training plans for species in conservation programs (Shepherdson, 1994; Reading et al., 2013; Michaels et al., 2014; Riley, 2018; Tetzlaff et al., 2019)—so too do humans have to adapt. Much literature, for example Ungar and Theron (2020) and DeRosier et al. (2013), can be found on the need for human populations to build resilience and strategies for coping with the stresses of modern living to promote mental wellbeing. The access to nature and immersion in green space that zoos provide has been shown to have a positive effect on human physical and psychological health (Coolman et al., 2020). Engagement with the zoo’s educational messaging enables pro-environmental human behavior change (Collins et al., 2020). The pro-environmental outcomes of zoo education and engagement programs can be measured using social science methods to provide a blueprint for the development of effective education strategies across organizations that evaluate long-term positive effects (Mellish et al., 2019). This can help develop the Wellbeing aim of the zoo further, if individual organizations have standardized ways of measuring the efficacy of their conservation education programs, they can implement the most relevant and impactful engagement or educational regime.

Wellbeing impacts spread outside of the zoo’s grounds too. For example, collaboration between zoo experts, and field-based natural and social scientists, combined with conservation funding from zoos for work in areas of emerging infectious diseases provides One Health benefits to communities, ecosystems and wildlife holistically (Robinette et al., 2017). An estimated $350 million is spent on conservation by World Association of Zoos & Aquariums members annually (Gusset and Dick, 2011), and as zoo visitation is influenced by (amongst other things) the richness and diversity of the animal collection (Mooney et al., 2020), so conservation income correlates with species diversity. Therefore, welfare of the living collection is an essential foundation to the successful fulfilment of CERR aims.

The essential foundation for the zoo’s impact and the outputs it wishes to achieve is the living collection of plants and animals that it houses. Further development of husbandry techniques, refinement and evaluation of environmental enrichment programs, and the measurement of individual behavior patterns over an individual’s lifetime sites welfare at the center of the zoo’s animal-focused operations. This is approach essential for encouraging repeat visits to the zoo, as visitors engaging with animals perceived to be “content” or “comfortable” or “natural” or even “happy” are more likely to leave with a positive overall impression of the zoo and its reasons for existing (Klenosky and Saunders, 2007).

## Evolving recreation into engagement

By including Wellbeing as a core aim of the modern zoo, the current focus on animal welfare improvements continues alongside of its role in upholding sustainable conservation, impactful education, and valid research outputs) and is aligned with newer ventures into the promotion of human wellbeing and One Health initiatives for conserving biodiversity and ecosystem functions. Whilst Recreation may be a clear goal of many zoos, as the main way in which revenue is earned to support Conservation, Education and Research (and Wellbeing?) aims, Recreation may be a label that suggests the work and activities of zoos are trivial or frivolous. A place only for entertainment and amusement. We propose that a better way of advancing the goals of the modern zoo, and to share its impact more widely is to suggest that Recreation at the zoo is Engagement. Engagement with the natural world and engagement with the Conservation, Education and Research outputs of the zoo.

It is essential that a zoo’s operations are financially viable and sustainable for the future (Liptovszky, 2020), as the ultimate care of the animals, employment of the staff, and meeting of Education, Conservation and Research aims depends on financial support. Therefore, zoos balance holding species that ensure consistent visitation against those that require urgent conservation action (Turley, 1999; Catibog-Sinha, 2008; Carr, 2016). Engaging zoo visitors with the financial needs of conservation can be achieved by exhibit design that connects the visitors to the ecology, behavior and pressures on the species in the wild (Conway, 2011). Such an approach can provide the visitor with an understanding of the financial needs of the zoo, and where their entry fee is being used.

When learning and the attainment of new information is fun and interesting, educational objectives are likely to succeed (Lucardie, 2014). The zoo is in a unique position to promote an interesting and enjoyable experience that provides educational opportunities. This enhances visitor Wellbeing and, by supporting operational and husbandry decisions that advance animal welfare, Engagement also provides a clear foundation for the Wellbeing aim of the modern zoo.

**Figure 2:**
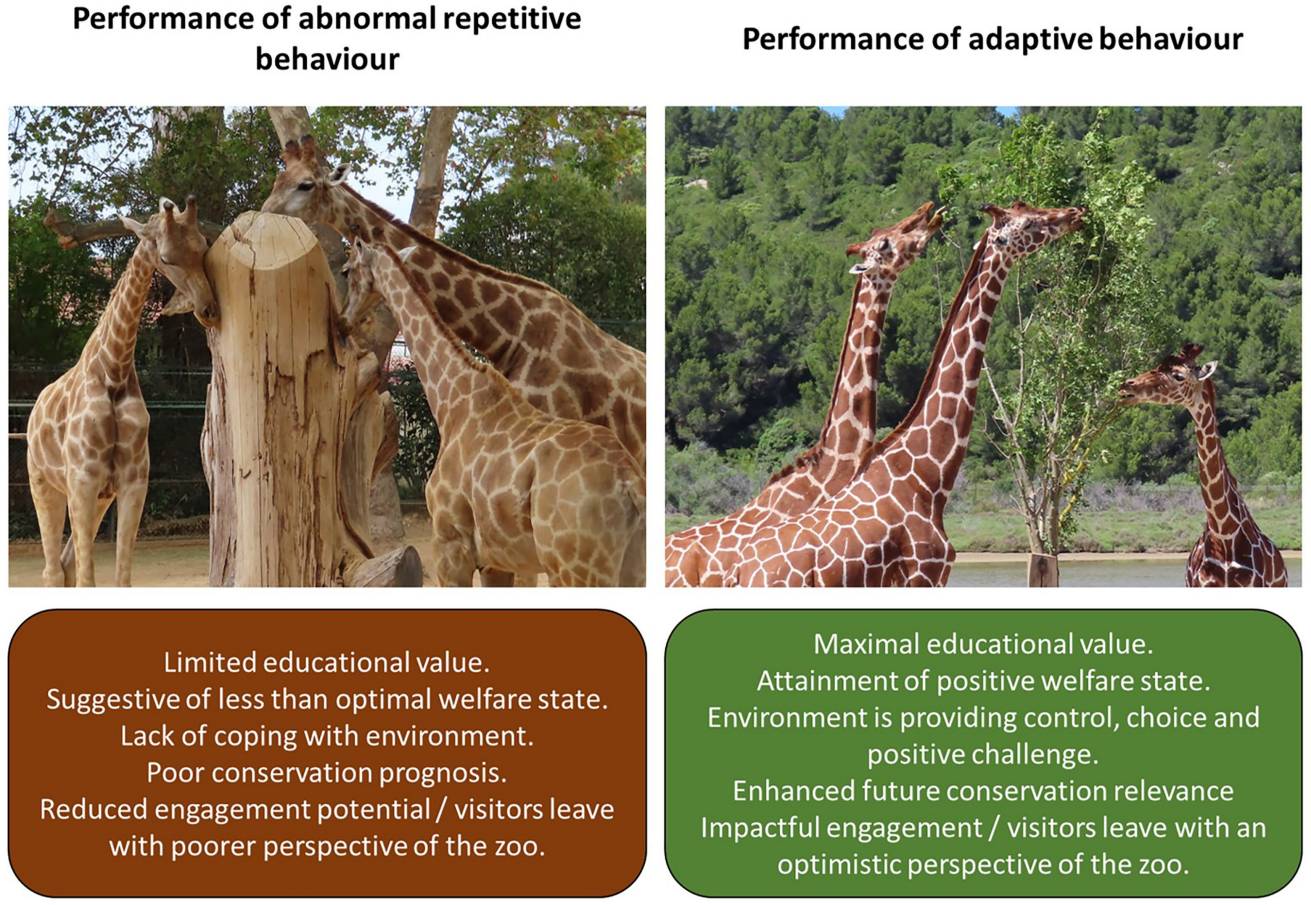
When visitors view abnormal repetitive behaviors, the key messages of the zoo as a place of education, science and conservation can be diluted. The Wellbeing aim of the zoo cannot be promoted if unnatural behavior patterns go unchecked. When animals have the opportunity to perform adaptive and highly motivated behaviors, it is clear to visitors that animal welfare is at the heart of the zoo’s mission and the other associated goals of the zoo are enhanced by the performance of animals being naturally. Examples of poorer giraffe welfare (vacuum chewing and licking against enclosure furnishings) compared to positive giraffe welfare (browse is provided regularly as a portion of the animal’s daily dietary intake).

Figure 2 illustrates the same species but with two very different connotations of animal welfare state. Giraffes (*Giraffa camelopardalis*) are prone to the development of abnormal repetitive behaviors (e.g., stereotypic oral actions such as licking, vacuum chewing and wind sucking) in the zoo (Bashaw et al., 2001; Baxter and Plowman, 2001). Such behaviors can negatively impact on animal health and are suggestive of poorer welfare and a lack of suitable husbandry and diet (Baxter and Plowman, 2001; Kulkarni, 2020). When provided with opportunities to browse on tree branches, giraffes reduce abnormal behavior performance (Bashaw et al., 2001) and therefore illustrate their evolutionary adaptations for foraging to zoo visitors. Changes to husbandry that improve behavioral outputs and therefore welfare, can improve the visitor’s perspectives of the zoo and how the visitor feels towards the animals that are being observed (Coe, 1985). The visitor becomes more engaged with the species and the “story” that the species tells about its habitat, place in the world and how it is impacted by human activities if the animal presents as a replica of what it can do in the wild state.

Whilst natural or species-typical behavior is only one aspect of animal welfare, it is an important one for zoos to consider. A clear link between visitor’s perception of animal welfare and the display of abnormal behavior is demonstrated; poorer perception of the animal’s wellbeing, less confidence in the zoo’s abilities to properly care for the animal and reduced support for the zoo overall are all evident when an abnormal repetitive behavior is being viewed (Miller, 2012). Training and positive human contact reduce stress responses in captive wild animals (Vasconcellos et al., 2016) and therefore developing the human-animal bonds, where relevant to a species, is another essential aspect of promoting good animal welfare to the zoo’s visitors.

If zoos are to compete with computer technology, television natural history programs and other forms of media that display animals out in the wild, the animals that the visitor comes to see at the zoo need to be representative of what this species “is” when viewed in its natural habitat, which is of course of intrinsic value to the animal also [as per *naturalness* (Fraser, 2008)]. The educational aims of the zoo are not promoted and may appear disingenuous if animals cannot perform species typical behavior, contrasting to the educational messaging of the zoo that refer to the ecology and evolution of the species in its wild state. Ultimately, an Engagement aim of the zoo encompasses the recreational aspects of being immersed in nature but places further emphasis on zoos to continue to develop and evolve husbandry standards to ensure that animals can reach for positive welfare and not have to cope by performing unwanted and inappropriate behavior patterns.

## Future research to further understand wellbeing as an aim of the zoo

**Figure 3:**
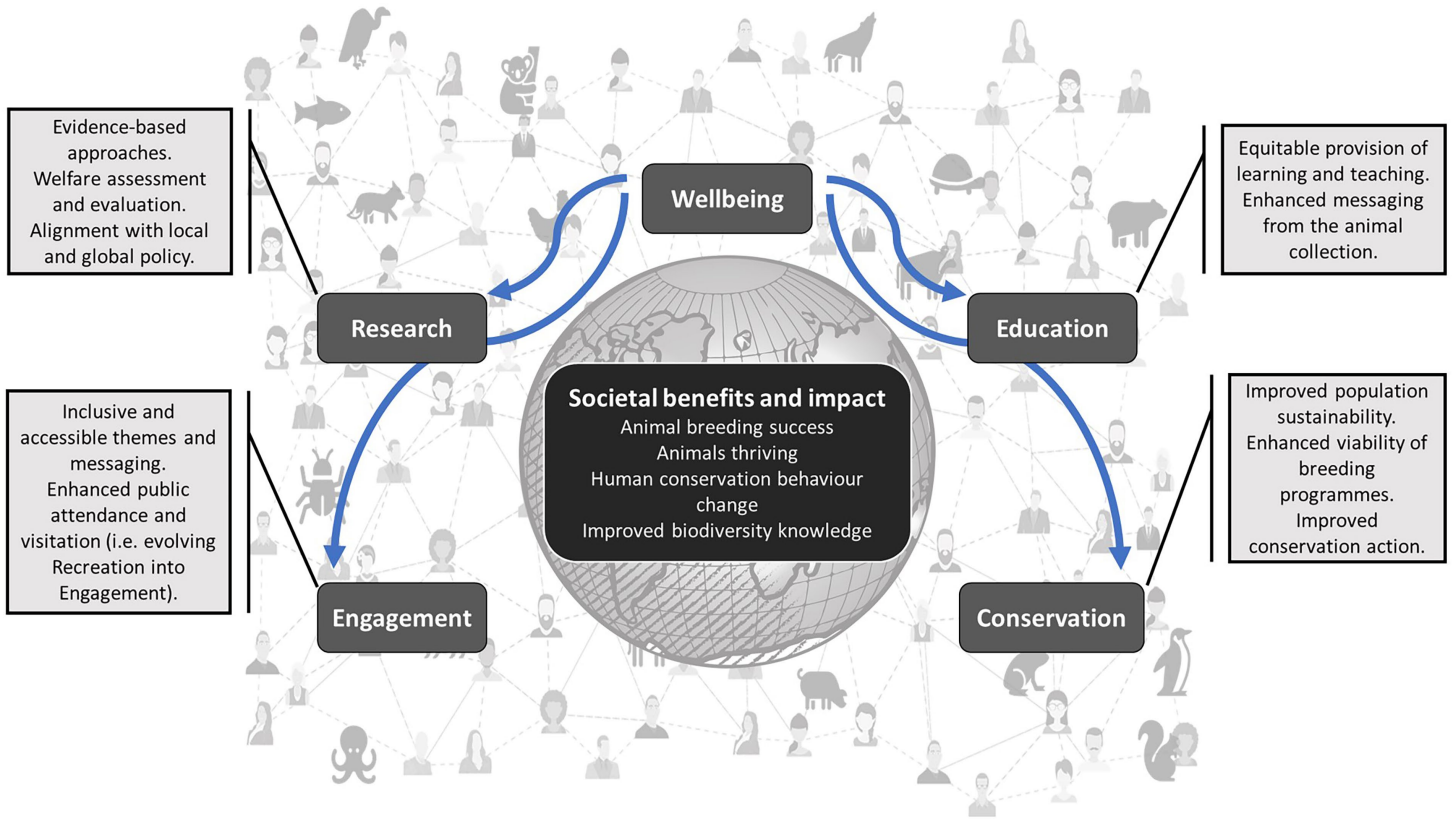
The benefits to humans and animals from including Wellbeing as the fifth aim of the modern zoo and aquarium. Suggested outputs that define the Engagement, Research, Education and Conservation aims of the zoo are placed alongside the wider potential impacts of the zoo when these all stem from a Wellbeing starting point.

The ideas presented in this paper support the Theory of Change presented in the Post-2020 Global Biodiversity Framework by encouraging zoos to implement a new aim for the benefit of humans and wildlife. As the Post-2020 Framework’s long term vision is to “live in harmony with nature by 2050” (Secretariat of the Convention on Biological Diversity, 2021) zoos can help push this ideal forward with a greater emphasis on Wellbeing and positive connection to the natural world. The diversity of a zoo’s workforce also provides emphasis for wellbeing to be given a key role because people from different socioeconomic and cultural backgrounds who are all connected to zoos will provide a multitude of ideas of how to better connect, and more meaningfully, with nature. Passive attempts at conservation messaging and education do not seem to leave a lasting impression on zoo visitors (Ojalammi and Nygren, 2018); by framing the aims and objectives of the zoo around animal welfare and human wellbeing, the conservation and education roles of the zoo may become more relevant to the daily lives of visitors and how their actions ultimately impact on planetary health and sustainability. Figure 3 provides an illustration of the interactions between the four current aims of the modern zoo, and the inclusion of the fifth Wellbeing aim.

Figure 3 summarizes the key points from this paper to show how Wellbeing is integral to the fulfilment of the other aims of the modern zoo by ultimately mandating wider societal benefits and impacts of zoo activities. For example, animals provided with appropriate environmental conditions that breed more successfully are better candidates for conservation breeding objectives. Improved sustainability and viability of many conservation breeding programs can be achieved with improvements to welfare that ultimately arise due to the implementation of species-relevant husbandry and management (Cikanek et al., 2014; Blais et al., 2022). Population management in zoos should consider the ultimate end-point of all individuals involved (Clay and Visseren-Hamakers, 2022), including the needs of those individuals within a managed population that may not be required for further or future conservation breeding needs (Carter and Kagan, 2010).

The human-animal relationship is also an important factor when considering the welfare of zoo-housed species (Cole and Fraser, 2018), and also plays a role in promoting positive wellbeing of zoo personnel too (Hosey and Melfi, 2012). Any Wellbeing aim of the modern zoo should consider the role of such inter-species bonds and the affect this relationship can have on positive (and negative) emotional states for both parties. Research has shown that zoo visitors place animal welfare one of the zoo’s top priorities (Roe et al., 2014). The behavior of the animals in the zoo is a key influence over visitor perception of their care and welfare (Salas et al., 2021). Animals that are viewed as thriving in the zoo impart more influence over the visitor’s experiences of the zoo and therefore are better tools to use to enact planetary friendly human behavior change. Ultimately, zoo-housed species that experience good welfare can be more useful to the zoo in imparting knowledge about biodiversity, how it is threatened and therefore what the zoo is doing to both directly and indirectly conserve it, and of course, each individual animal has a more positive lived experience and can thus experience good welfare. Zoo science and research departments can implement empirical programs of study to define the impact of such a Wellbeing aim on the humans and animals within the zoo, and to assess the wider impact of the zoo on society. Social science outputs from zoos are increasing and when combined with assessments of animal welfare would provide a useful holistic view of how Wellbeing is embedded into the zoo’s mission, values, and outputs. Table 1 provides some suggestions for topics and their impact that are relevant to future Wellbeing-focused research.

**Table 1:** Examples of topics (left-hand column) that could be crafted into research questions to measure, and then evidence, the impact of Wellbeing aims of the modern zoo (right-hand column).

Topic for future research	Potential impact
Fact finding *“The ability of zoo visitors to recall key information on animals, habitats or biomes is enhanced by the display of and engagement with the living collection.”*	Improves the welfare of the animals by encouraging the zoo to further develop species-appropriate husbandry that support species’ needs. Measuring long-term recall of key information and how this is improved by zoo visiting would evidence efficacy of zoo education outputs.
Small scale behavior change *“A greater understanding of sustainable shopping habitats and choice of products due to engagement with in-zoo messaging and storytelling around climate change”.*	Improves human wellbeing and animal welfare by encouraging planetary friendly, sustainable, behaviors. Measuring the number of zoo visitors that change behavior to improve their own and planetary wellbeing post-engagement with zoo educational messaging can evidence how to display behavior change information in the future.
Long-term behavior change *“Greater value given to nature and a deeper appreciation of the benefits of the natural world by visiting the zoo and regularly supporting its work.”*	Improves human wellbeing and animal welfare by encouraging planetary friendly, sustainable behaviors. Measuring how people change routines or previously entrenched activities post-zoo visit to deliver longer-term wellbeing benefits can evidence the benefits of regular, versus sporadic, zoo visiting and provide suggestions for how zoos encourage repeat visits.
Valuing local nature *“The importance of the zoo’s green/blue spaces to human health is embedded in the psyche of the zoo’s visitors and therefore is viewed as important on par with visiting to see the living collection.”*	Human wellbeing is promoted by regular access to nature and animal welfare is improved due to the added value placed on the living collection as a tool to enhance connectivity with the natural world. Measurement of psychological (e.g., improved mood) and physiological (e.g., lowered glucocorticoids or reduced heart rate) benefits evidences the importance of the future conservation of zoo green spaces.
Consideration of biodiversity *“Enhanced understanding of the role of the ex situ population to the conservation needs of free-living animal and wild places”.*	Improves animal welfare by encouraging the zoo to display species in a manner that increases engagement with wild-world conservation work.
Advocacy *“Lobbying of local government representative or similar to gain traction for environmentally-beneficial policies is increased due to increased education post-zoo visiting.”*	Improves human wellbeing and animal welfare by advocating for planetary-friendly policy change.

Wellbeing can ultimately go beyond supporting the Education aim for zoo visitors and can promote more than the welfare of the zoo’s animals or be a green prescribing platform for visitors. The actions of the zoo itself, for example using sustainable energy sources, conserving water, and considering the carbon footprint of goods and services used, all would promote planetary wellbeing and the associated positive impacts on humans and wildlife. “Action by doing” could be promoted by a trip to the zoo as visitors can leave with ideas of how to be sustainable in their own lives having viewed such initiatives during their time at the zoo (Mann et al., 2018; Routman et al., 2022). Alongside of visitors, the wellbeing of zoo staff can be promoted by Wellbeing becoming a key zoo aim. Proactive measures from zoo management to combat compassion fatigue (Figley and Roop, 2006; Hill et al., 2020), for example, will not only benefit the health and wellbeing of personnel but will eventually promote good animal welfare by ensuring staff feel capable of executing their role as expertly as possible (Yam et al., 2022). When zoo staff feel valued and invested in, and they are provided with the relevant skills and tools needed, they can better implement species-relevant animal husbandry that is the foundation of positive animal welfare in the zoo.

Evaluating how the zoo meets these aims is important and research that quantifies how such aims are met and by how many member/accredited zoos would be useful. Although the outputs from zoos are expansive, particularly in the fields of scientific research pertaining to animal husbandry, behavior and ecology (Loh et al., 2018; Rose et al., 2019; Hvilsom et al., 2020; Escribano et al., 2021), assessment of how conservation objectives (Escribano et al., 2021) and education objectives (Moss and Esson, 2013) are effectively met by zoos is still required. A broader review of how zoos define and examine learning outcomes across different demographic groups would help further assess coverage of Education aims (Schilbert and Scheersoi, 2022). These authors go on to state that the nurturing of pro-environmental actions and behaviors in their visitors is a common goal of zoos. And even if limitations in how such ideals are measured evaluated are noted, zoos have the potential to contribute greatly to both conservation education and biodiversity conservation initiatives (Schilbert and Scheersoi, 2022). Studies have also revealed that biodiversity-centered knowledge can be increased (over the long term) from experiences at the zoo (Jensen et al., 2017). Consequently, a cyclical process of reflection and examination of the aims of the zoo, and how they are met, is required to ensure they remain relevant. Adding in Wellbeing as a further, measurable, aim of the zoo and evolving Recreation into Engagement may help differentiate between zoos that have evidence for how the aims of the modern zoo are met, compared to those that fall short.

## Conclusion

Modern zoos evidence impactful work in the fields of conservation and research and provide a point of interest and a focus for public engagement for many thousands of visitors annually. The four current aims of the modern zoo provide clear explanation of the past efforts of zoos and how they have attempted to conserve and protect biodiversity for the past 40 years. To further move the work of zoos into the 21^st^ century, the fifth aim of the future zoo should be one to promote wellbeing. Enhancing animal lives and conservation messaging and opportunities for human behavior change, which are (in turn) supported by a properly cared for living collection that uses evidence to inform practice. Engagement is a fundamental component of a zoo’s Recreation aim and engaging the audience with positive wellbeing *via* access to animals and green space increases the value of the visit to the zoo. Including Wellbeing and changing Recreation to Engagement further strengthens the zoo’s societal, cultural, and global reach and adds value to the existence of the living collection. By bringing together positive aspects of human mental health—considering the value of animal collections as green, outdoor spaces that allow for connection with nature, as well as how positive animal welfare states can be maintained that evidence good care of the species housed—zoo operations become more sustainable with wider benefits to humans and animals.

## Author contributions

All authors listed have made a substantial, direct, and intellectual contribution to the work and approved it for publication.

## Conflict of interest

The authors declare that the research was conducted in the absence of any commercial or financial relationships that could be construed as a potential conflict of interest.

## Publisher’s note

All claims expressed in this article are solely those of the authors and do not necessarily represent those of their affiliated organizations, or those of the publisher, the editors and the reviewers. Any product that may be evaluated in this article, or claim that may be made by its manufacturer, is not guaranteed or endorsed by the publisher.
